# Mediators of SARS-CoV-2 entry are preferentially enriched in cardiomyocytes

**DOI:** 10.1186/s41065-020-00168-4

**Published:** 2021-01-04

**Authors:** Jing Yang, Tan Chen, Yafeng Zhou

**Affiliations:** 1 Department of Cardiology, The First Affiliated Hospital of Soochow University, Suzhou, 215006 China; 2 Department of Cardiology, Dushuhu Public Hospital Affiliated to Soochow University, Suzhou, 215000 China

**Keywords:** SARS-CoV-2, COVID-19, ACE2, CTSB, CTSL, cardiac injury

## Abstract

**Background:**

The coronavirus disease 2019 (COVID-19) has spread rapidly around the world. In addition to common respiratory symptoms such as cough and fever, some patients also have cardiac injury, however, the mechanism of cardiac injury is not clear. In this study, we analyzed the RNA expression atlases of angiotensin-converting enzyme 2(ACE2), cathepsin B (CTSB) and cathepsin L (CTSL) in the human embryonic heart at single-cell resolution.

**Results:**

The results showed that ACE2 was preferentially enriched in cardiomyocytes. Interestingly, serine protease transmembrane serine protease 2 (TMPRSS2) had less expression in cardiomyocytes, but CTSB and CTSL, which belonged to cell protease, could be found to be enriched in cardiomyocytes. The results of enrichment analysis showed that differentially expressed genes (DEGs) in ACE2-positive cardiomyocytes were mainly enriched in the processes of cardiac muscle contraction, regulation of cardiac conduction, mitochondrial respiratory chain, ion channel binding, adrenergic signaling in cardiomyocytes and viral transcription.

**Conclusions:**

Our study suggests that both atrial and ventricular cardiomyocytes are potentially susceptible to severe acute respiratory syndrome coronavirus-2(SARS-CoV-2), and SARS-CoV-2 may enter ventricular cardiomyocytes using CTSB/CTSL for S protein priming. This may be the partial cellular mechanism of cardiac injury in patients with COVID-19.

## Background

In December 2019, some cases of pneumonia with unknown etiology occurred in Wuhan, Hubei Province, China [1]. The new pneumonia spread rapidly in China. Subsequently, the World Health Organization announced that this new epidemic disease caused by severe acute respiratory syndrome coronavirus-2(SARS-CoV-2) is the coronavirus disease 2019 (COVID-19) [2]. Now COVID-19 has spread around the world, as of November 13, 2020, COVID-19 has caused 52, 736, 076 confirmed infections and 1, 293, 192 deaths worldwide (https://coronavirus.jhu.edu/map.html). In addition to common respiratory symptoms such as cough and fever, some patients may also have diarrhea and liver damage [3].

It has been established that patients with COVID-19 have well-documented cardiac complications [4]. A recent study showed that 19.7% of patients with COVID-19 had cardiac injury, which was associated with higher risk of in-hospital mortality [5].However, the mechanism of cardiac injury is not clear. COVID-19 is caused by SARS-CoV-2, which belongs to Betacoronavirus and is an enveloped and positive-sense single-stranded RNA (+ssRNA) virus [6].SARS-CoV-2 mainly uses angiotensin-converting enzyme 2(ACE2) as the entry receptor [7], and it employs the cellular serine protease transmembrane serine protease 2(TMPRSS2) for spike(S) protein priming [8]. In some cases, SARS-CoV-2 could use cathepsin B (CTSB) or cathepsin L (CTSL) entering TMPRSS2-negative cells [9], which means that cathepsin B/L has the potential to replace TMPRSS2 in function.

In this study, we analyzed the RNA expression atlases of ACE2, CTSB and CTSL in the human embryonic heart at single-cell resolution. The results showed that ACE2 is mainly enriched in cardiomyocytes. Interestingly, serine protein TMPRSS2 had less expression in cardiomyocytes, but CTSB and CTSL, which belonged to cell protease, could be found to be enriched in cardiomyocytes. The differentially expressed genes (DEGs) in ACE2-positive cardiomyocytes were mainly enriched in the processes of cardiac muscle contraction, regulation of cardiac conduction, mitochondrial respiratory chain, ion channel binding, adrenergic signaling in cardiomyocytes and viral transcription. Our study suggests that cardiomyocytes have a potential susceptibility to SARS-CoV-2, which may be one of the cellular mechanisms of cardiac injury in patients with COVID-19.

## Methods

The 10x genomics scRNA seq data of human embryo was from Single Cell Expression Atlas (https://www.ebi.ac.uk/gxa/sc/home, accessed 2 May 2020), and the expression atlas accession number of scRNA seq data was E-HCAD-7. 8–11 week human embryonic heart data was selected from the total Single-cell RNA sequencing (scRNA-seq) data.and the selected 10x genomics scRNA-seq data was converted into hdf5 object handled by scanpy on galaxy website (https://humancellatlas.usegalaxy.eu/tours). Then we imported scanpy object into BBrowser2 software (version:2.4.10) for scRNA-seq data bioinformatics analysis. Cells with less than 200 genes and more than 5% mitochondrial genes were excluded, and only the first 2000 variable genes were used for subsequent analysis. Different cell types were identified by mapping canonical marker genes in the t-distributed stochastic neighbor embedding (tSNE) plot, and marker genes of different cell clusters were shown in the heat map. Finally, the DEGs of ACE2-positive cardiomyosytes were identified compared with ACE2-negative cardiomyocyte in BBrowser2 software (version:2.4.10). |log FC| > 1 and *P* value < 0.05 were set as the cutoff criteria for DEGs. Gene ontology and pathway enrichment analyses of DEGs were further performed in DAVID website (https://david.ncifcrf.gov/) [10].

## Results

### Identification of cell types in human heart

**Fig. 1:**
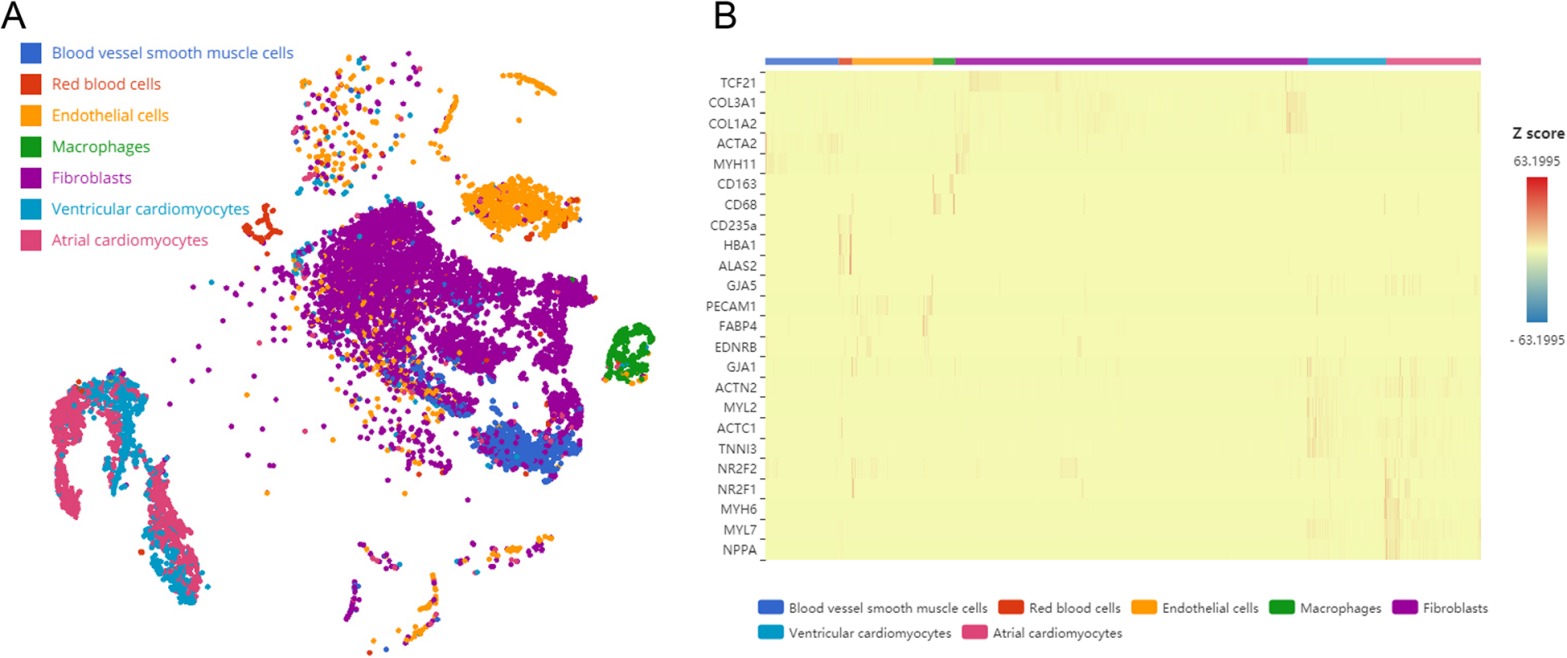
Single-cell RNA sequencing analysis of human heart. **a** t-distributed stochastic neighbor embedding (tSNE) plot for different cell types from heart samples. **b** Heatmap of differentially expressed marker genes for each clusters

We analyzed the human embryonic heart data, which contains normal human embryonic heart tissues.11512 cells were used for subsequent analysis after quality control, tSNE and marker genes analyses were performed in these cells.7 major cell clusters had been identified (Fig. 1a). We annotated the cell clusters according to the classical marker genes from previous literatures [11, 12] and CellMarker database (http://biocc.hrbmu.edu.cn/cell marker/index. JSP) (Fig. 1b), these cell clusters included blood vessel smooth muscle cells, red blood cells, endothelial cells, macrophages, fibroblasts, ventricular cardiomyocytes and atrial cardiomyocytes.

### Cell type-specific expression atlas of mediators of SARS-CoV-2 entry in human heart

**Fig. 2:**
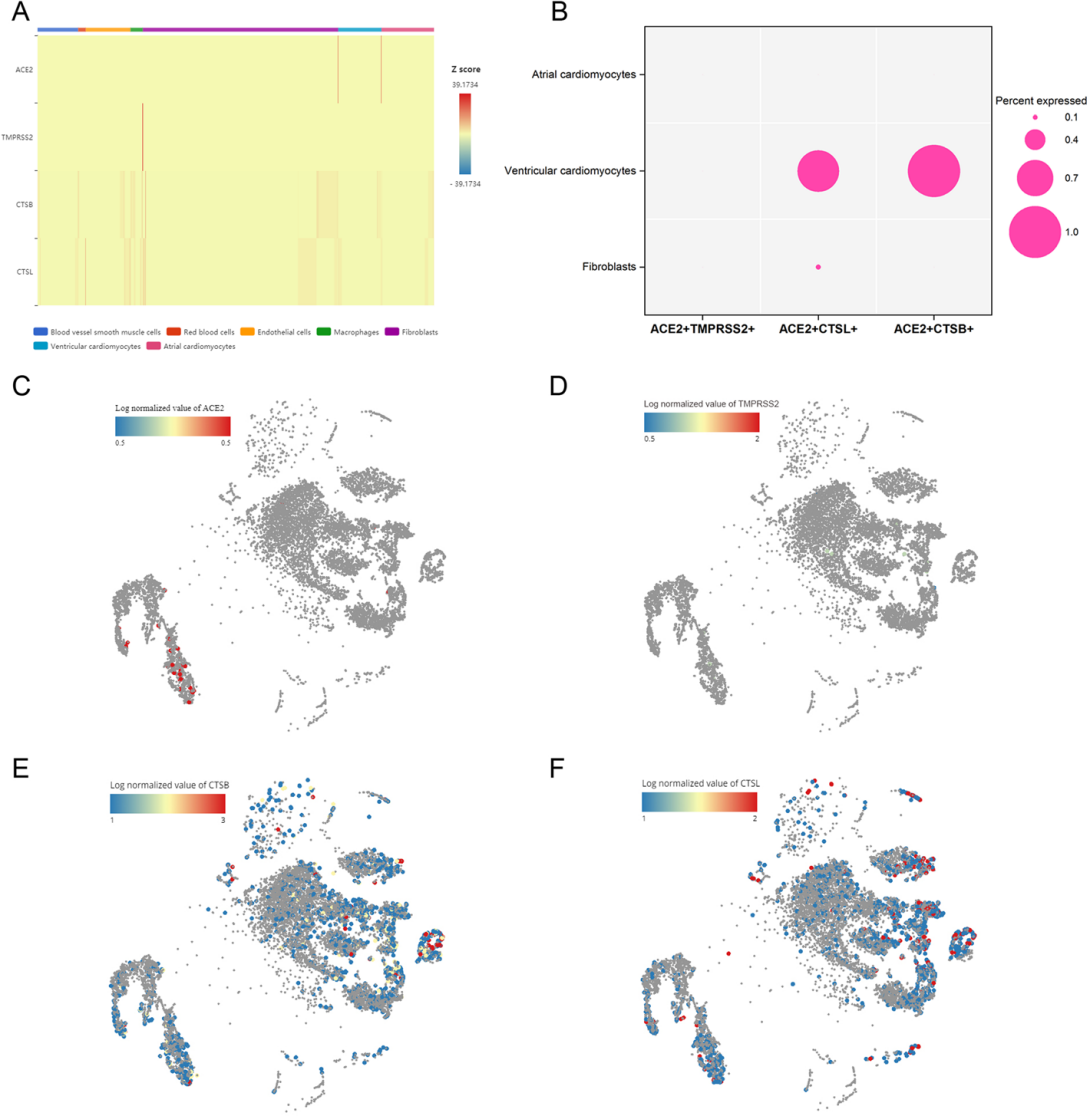
Mediators of SARS-CoV-2 entry were expressed by cardiomyocytes. **a** ACE2, TMPRSS2, CTSB and CTSL expression across different cell types. **b** Percentages of double positive cells in fibroblasts, ventricular cardiomyocytes and atrial cardiomyocytes. **c**-**f** t-distributed stochastic neighbor embedding (tSNE) maps indicating the expression of ACE2, TMPRSS2, CTSB and CTSL in identified cell populations

For the analysis of mediators of SARS-CoV-2 entry in human heart, we compared the expression of ACE2, TMPRSS2, CTSB and CTSL in different cell clusters. The results showed that ACE2 was specifically expressed in cardiomyocytes and fibrocytes (Fig. 2a, c), but there was a higher expression of ACE2 in cardiomyocytes than fibrocytes. Interestingly, serine protein TMPRSS2 had less expression in cardiomyocytes, but CTSB and CTSL, which belonged to cell protease, could be found to be enriched in cardiomyocytes (Fig. 2a, d-f). We further explored the proportion of double positive cells in cardiomyocytes and fibrocytes. We found that no cell cluster contained ACE2 + TMPRSS2+ cells. However, ventricular cardiomyocytes contained more ACE2 + CTSL+ cells (0.8%) and ACE2 + CTSB+ cells (1%), small proportions of ACE2 + CTSL+ cells (0.1%) were found in fibroblasts, and there were no double positive cells in the atrial cardiomyocytes (Fig. 2b). A recent study showed that SARS-CoV-2 mainly used ACE2 receptor to enter host cells and employed cellular serine protease TMPRSS2 for S protein priming [8].However, SARS-CoV-2 can also use CTSB/CTSL entering TMPRSS2- cells [9], this means that CTSB/CTSL has the potential to replace TMPRSS2 in function. Taken together, these findings suggest that both ventricular cardiomyocytes and atrial cardiomyocytes are potentially susceptible to SARS-CoV-2.It is worth mentioning that SARS-CoV-2 may enter ventricular cardiomyocytes by using ACE2 and CTSB/CTSL.

### Gene ontology and pathway enrichment analysis of DEGs in ACE2-positive cardiomyocytes

**Fig. 3:**
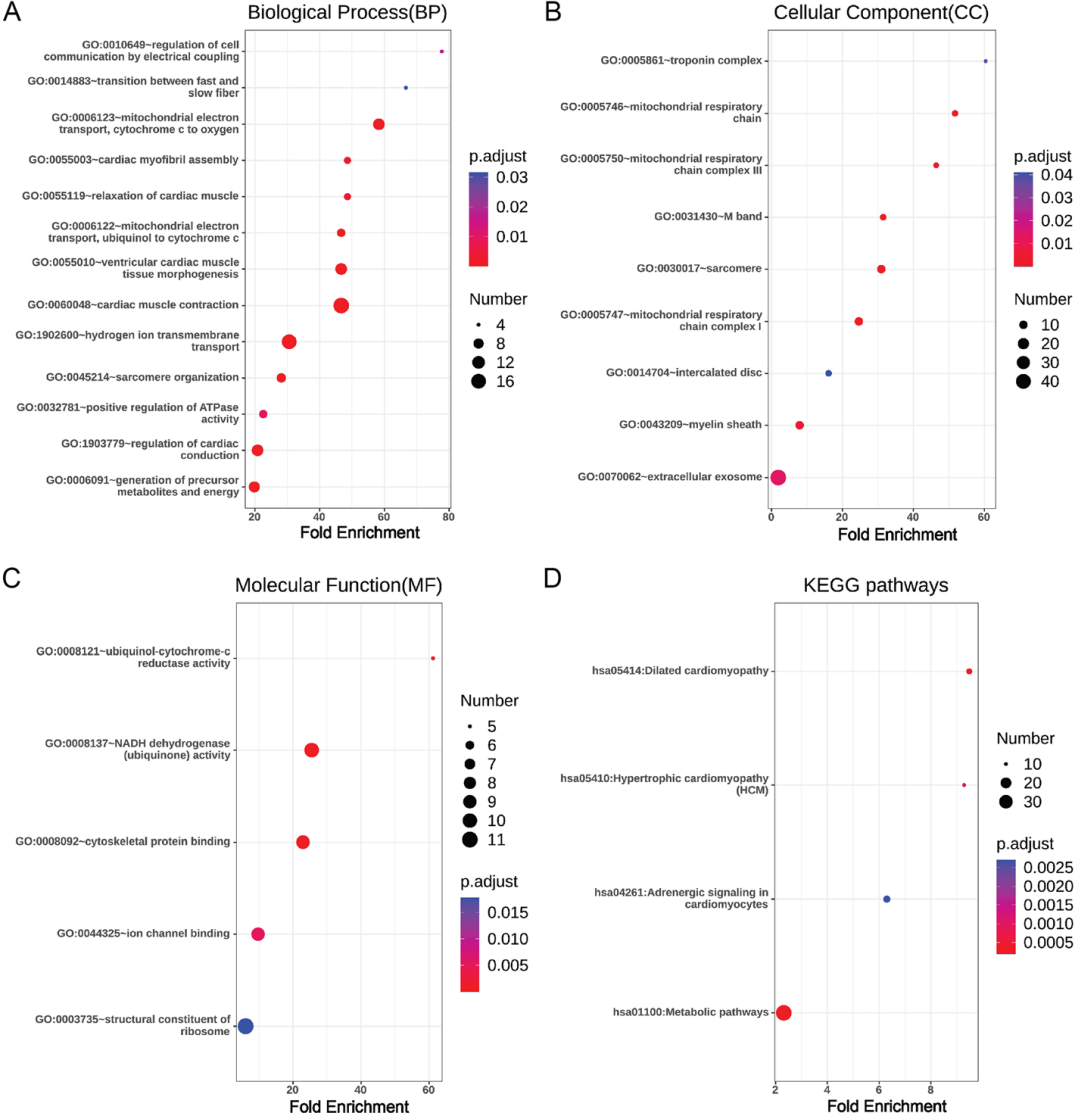
Enrichment analysis of up-regulated DEGs in ACE2-positive ventricular cardiomyocytes. **a**: Biological Process, **b** Cellular Component, **c** Molecular Function, and **d** KEGG pathways were presented. The size of the dot represents the counts of enriched up-regulated DEGs, and the color of the dot represents the adjusted *p* value

There were 177 up-regulated DEGs and 33 down-regulated DEGs in ACE2-positive ventricular cardiomyocytes, and 190 up-regulated DEGs and 18 down-regulated DEGs in ACE2-positive atrial cardiomyocytes. Gene ontology and pathway enrichment analysis of DEGs were performed by comparing ACE2-positive cells with ACE2-negative cells in ventricular cardiomyocytes and atrial cardiomyocytes. The biological process, cellular component, molecular function and KEGG pathways enrichment results of up-regulated DEGs in ACE2-positive ventricular cardiomyocytes were presented in Fig. 3. It could be found that up-regulated DEGs were particularly enriched in cardiac muscle contraction, hydrogen ion transmembrane transport, mitochondrial electron transport, cytochrome c to oxygen, ventricular cardiac muscle tissue morphogenesis, regulation of cardiac conduction, generation of precursor metabolites and energy, mitochondrial electron transport, ubiquinol to cytochrome c, sarcomere organization, relaxation of cardiac muscle, cardiac myofibril assembly, positive regulation of ATPase activity, regulation of cell communication by electrical coupling and transition between fast and slow fiber for biological process (Fig. 3a), sarcomere, mitochondrial respiratory chain complex I, mitochondrial respiratory chain, M band, mitochondrial respiratory chain complex III, myelin sheath, extracellular exosome, troponin complex and intercalated disc for cellular component (Fig. 3b), NADH dehydrogenase (ubiquinone) activity, cytoskeletal protein binding, ubiquinol-cytochrome-c reductase activity, ion channel binding and structural constituent of ribosome for molecular function (Fig. 3c), and metabolic pathways, dilated cardiomyopathy, hypertrophic cardiomyopathy and adrenergic signaling in cardiomyocytes for KEGG pathways (Fig. 3d). In addition, the down-regulated DEGs were particularly enriched in extracellular matrix organization, extracellular exosome, protein binding and structural constituent of cytoskeleton (Table 1).

**Table 1:**
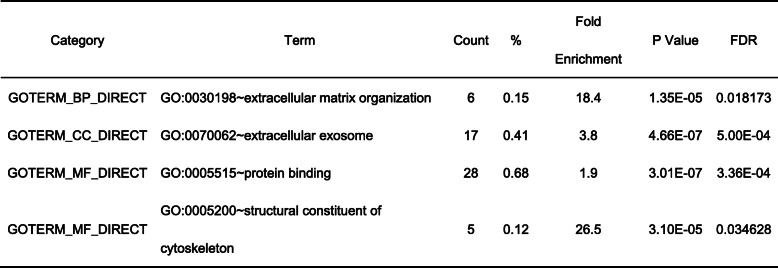
Enrichment analysis of down-regulated DEGs in ACE2-positive ventricular cardiomyocytes

*BP* Biological Process, *CC* Cellular Component, *MF* Molecular Function

**Fig. 4:**
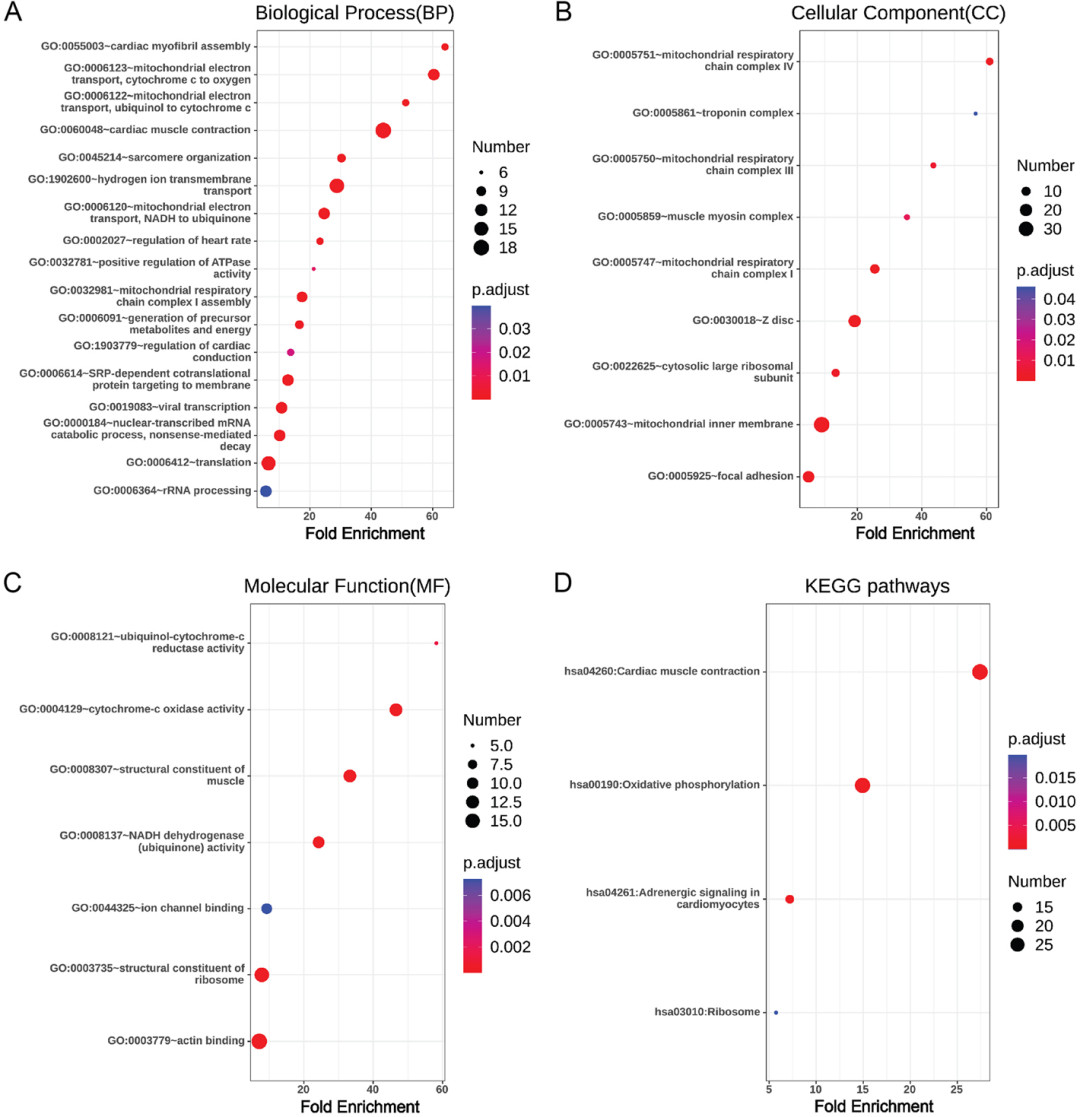
Enrichment analysis of up-regulated DEGs in ACE2-positive atrial cardiomyocytes. **a** Biological Process, **b** Cellular Component, **c** Molecular Function, and **d** KEGG pathways were presented. The size of the dot represents the counts of enriched up-regulated DEGs, and the color of the dot represents the adjusted *p* value

Besides, the biological process, cellular component, molecular function and KEGG pathways enrichment results of up-regulated DEGs in ACE2-positive atrial cardiomyocytes were presented in Fig. 4.We found that up-regulated DEGs were enriched in viral transcription, regulation of heart rate, regulation of cardiac conduction, cardiac muscle contraction, hydrogen ion transmembrane transport, mitochondrial electron transport, cytochrome c to oxygen, mitochondrial electron transport, NADH to ubiquinone, cardiac myofibril assembly, mitochondrial electron transport, ubiquinol to cytochrome c, mitochondrial respiratory chain complex I assembly, sarcomere organization, SRP-dependent cotranslational protein targeting to membrane, translation, nuclear-transcribed mRNA catabolic process, nonsense-mediated decay, generation of precursor metabolites and energy, positive regulation of ATPase activity and rRNA processing for biological process (Fig. 4a), mitochondrial inner membrane, Z disc, mitochondrial respiratory chain complex I, mitochondrial respiratory chain complex IV, focal adhesion, cytosolic large ribosomal subunit, mitochondrial respiratory chain complex III, muscle myosin complex and troponin complex for cellular component (Fig. 4b), cytochrome-c oxidase activity, structural constituent of muscle, NADH dehydrogenase (ubiquinone) activity, actin binding, structural constituent of ribosome, ubiquinol-cytochrome-c reductase activity and ion channel binding for molecular function (Fig. 4c), and cardiac muscle contraction, oxidative phosphorylation, adrenergic signaling in cardiomyocytes and ribosome for KEGG pathways (Fig. 4d).However, down-regulated DEGs in ACE2-positive atrial cardiomyocytes had no significant enrichment results.

## Discussion

Infectious diseases such as the Middle East respiratory syndrome-related coronavirus (MERS-CoV) can cause heart failure and acute myocarditis [13]. As the pathogen of COVID-19, SARS-CoV-2 has similar pathogenicity to MERS-CoV, the cardiac injury related to SARS-CoV-2 has attracted more and more attention. However, the mechanism of cardiovascular injury caused by COVID-19 has not been fully elucidated, which may be the result of multiple factors [14].

Here we reported the RNA expression atlases of ACE2 at the single-cell resolution in human embryonic heart. We found that ACE2 was preferentially enriched in cardiomyocytes compared with other types of cells in the heart. Interestingly, serine protein TMPRSS2 had less expression in cardiomyocytes, but CTSB and CTSL, which belonged to cell protease, could be found to be enriched in cardiomyocytes. We further explored the proportion of double positive cells in cardiomyocytes and fibroblasts, the results showed that ACE2 + CTSB+ cells and ACE2 + CTSL+ cells were mainly in the ventricular cardiomyocytes, and there were a few ACE2 + CTSL+ cells in the fibroblasts, but the double positive cells were not found in the atrial cardiomyocytes. It suggests that both atrial and ventricular cardiomyocytes are potentially susceptible to SARS-CoV-2, and SARS-CoV-2 may enter ventricular cardiomyocytes using CTSB/CTSL for S protein priming. Burrell, et al. found that the expressions of ACE2 mRNA in the viable myocardium of rats with myocardial infarction increased compared with the control group, and the immunoreactivity of ACE2 increased in the failing human heart [15]. Goulter, et al. found that the expression levels of ACE2 mRNA in idiopathic dilated cardiomyopathy and ischemic cardiomyopathy were significantly higher than that in non-diseased myocardium [16]. Mehra, et al. created a doxorubicin-induced rat model of cardiotoxicity to mimic human dilated cardiomyopathy and observed significant increases in CTSL activity and protein expression levels in rats treated with doxorubicin compared with the control group [17]. The expression levels of ACE2 and CTSL increase in the pathological state of the heart, which creates a favorable condition for the SARS-CoV-2 to invade the heart. Therefore, we need to pay special attention to COVID-19 patients with myocardial infarction or cardiomyopathy. These patients may experience severe SARS-CoV-2-induced cardiac injury due to the high expressions of ACE2 or CTSL in the diseased heart. It may be necessary to take preventive measures for patients with this complicated situation to avoid cardiac injury.

The gene ontology and pathway enrichment analysis of DEGs in ACE2-positive cardiomyocytes were performed, it showed that the DEGs in ACE2-positive ventricular cardiomyocytes were mainly enriched in cardiac muscle contraction, hydrogen ion transmembrane transport, mitochondrial electron transport, regulation of cardiac conduction, mitochondrial respiratory chain, NADH dehydrogenase (ubiquinone) activity, ion channel binding, metabolic pathways, adrenergic signaling in cardiomyocytes, extracellular exosome and other processes. Similarly, the DEGs in ACE2-positive atrial cardiomyocytes were mainly enriched in viral transcription, regulation of heart rate, regulation of cardiac conduction, cardiac muscle contraction, NADH to ubiquinone, mitochondrial respiratory chain complex I, mitochondrial respiratory chain complex IV, NADH dehydrogenase (ubiquinone) activity, actin binding, ion channel binding for molecular function, oxidative phosphorylation, adrenergic signaling in cardiomyocytes and other processes. Because ACE2-positive cardiomyocytes are the main targets of SARS-CoV-2, the damage of these cells may lead to the dysfunction of cardiac muscle contraction ability, cardiac electrical conduction process and intracellular mitochondrial respiratory chain. It may be the partial cellular mechanism of cardiac injury in patients with COVID-19.

In addition, patients with COVID-19 can experience cytokine storms when their condition is severe, which can damage myocardium [18]. A study related COVID-19 reported that 89.9% of patients with COVID-19 took antiviral drugs [1], which might also cause cardiac injury because many antiviral drugs could cause cardiac insufficiency. And the proposed mechanism of cardiac injury may also include myocardial interstitial fibrosis, interferon-mediated immune response, excessive cytokine response of type 1 and type 2 helper T cells, coronary plaque instability, and hypoxia [19]. There are also some limitations in our study, first of all, the results of this study are based on the analysis of human embryonic heart single-cell sequencing data, therefore, caution is needed when it is applied to the human adult heart. But a recent study showed that the expression levels of ACE2 in cardiomyocytes increased during the aging process of monkey hearts, but the proportion of ACE2^+^ cells remained unchanged in the cardiomyocytes of young and old monkeys [20]. It suggests that human adult cardiomyocytes may have a higher expression levels of ACE2, which contributes to the invasion of SARS-CoV-2 into cardiomyocytes. So our data indicate a general direction that needs to be further confirmed in the human adult heart. Secondly, only the RNA expression of ACE2, TMPRSS2, CTSB and CTSL were analyzed, their protein expression levels were ambiguous. Finally, this study is a bioinformatics analysis, and in-depth experimental verification is urgently required in the future.

## Conclusions

In summary, ACE2 was preferentially enriched in cardiomyocytes compared with other types of cells in the heart. Serine protein TMPRSS2 had less expression in cardiomyocytes, but CTSB and CTSL, which belonged to cell protease, could be found to be enriched in cardiomyocytes. It suggests that both atrial and ventricular cardiomyocytes are potentially susceptible to SARS-CoV-2, and SARS-CoV-2 may enter ventricular cardiomyocytes using CTSB/CTSL for S protein priming. This may be the partial cellular mechanism of cardiac injury in patients with COVID-19.

## Data Availability

The datasets used and/or analysed during the current study are available from the corresponding author on reasonable request.

## Abbreviations

SARS-CoV-2: Severe acute respiratory syndrome coronavirus-2
COVID-19: The coronavirus disease 2019
+ssRNA: Positive-sense single-stranded RNA;ACE2, angiotensin-converting enzyme 2
TMPRSS2: Serine protease transmembrane serine protease 2
CTSB: Cathepsin B
CTSL: Cathepsin L
DEGs: Differentially expressed genes
tSNE: t-distributed stochastic neighbor embedding
MERS-CoV: the Middle East respiratory syndrome-related coronavirus
